# Trends and Disparities in Non-fatal Firearm Injuries among Working-Age Adults in the United States, 2000–2021

**DOI:** 10.1007/s10900-024-01431-9

**Published:** 2024-12-19

**Authors:** Akshaya Bhagavathula, James H. Price, Jagdish Khubchandani

**Affiliations:** 1 Department of Public Health, North Dakota State University, Fargo, ND 58102 USA; 2 Division of Population Health, University of Toledo, Toledo, OH 43606 USA; 3 Department of Public Health Sciences, New Mexico State University, Las Cruces, NM 88003 USA

**Keywords:** Firearms, Injuries, Violence, Trauma, Risk, Epidemiology

## Abstract

Firearm-related injuries remain a significant public health issue in the United States, with patterns and trends among various age groups not well characterized. This study analyzed time series trends and disparities in firearm injury rates among U.S. working-age adults from 2000 to 2021. A retrospective analysis using data from the National Electronic Injury Surveillance System-Firearm Injury Surveillance Study (NEISS-FISS) was conducted with a focus on non-fatal firearm injuries reported in emergency departments across a nationally representative sample of hospitals. Descriptive statistics were used to explore disparities across different demographic groups. Trends were assessed using locally estimated scatterplot smoothing (LOESS) and Joinpoint regression analysis. Between 2000 and 2021, an estimated 2.36 million non-fatal firearm injuries occurred, with males accounting for 85.7% and non-Hispanic Blacks (NHB) representing 48.8% of injuries. Firearm injuries were commonly associated with crime (29.8%), physical fights (24.7%), alcohol/substance use (17.7%), and verbal arguments (17.2%). Most incidents occurred on weekdays (63.7%) and assaults were the most prevalent intent (68.5%), followed by unintentional injuries (21.9%). Handguns (25.1%) and unspecified firearm types (61.2%) were the most commonly involved weapons. From 2000 to 2021, significant increases in firearm injuries were observed among those aged 26–45 years (23.0%), women (21.97%), NHB (42.15%), and those involving assaults (231.9%). Age-specific trends showed a significant annual percentage change (APC) increase of 4.9% for 18–25 years, 12.4% for 26–45 years, and 7.0% for 46–64 years from 2013 to 2021. Racial/ethnic trends revealed a significant APC increase of 5.0% for Non-Hispanic Whites (2014–2021), 25.0% for NHB (2015–2021), and a decrease of -31.3% followed by an increase of 15.6% for Hispanics (2012–2021). The disproportionate burden of firearm injuries among different age and racial/ethnic groups highlights the need for targeted prevention strategies and ongoing monitoring of injuries.

## Background

Firearm violence is a significant and ongoing problem in the United States (U.S.), with serious consequences for public health and societal well-being. The U.S. has one of the highest rates of firearm violence among developed nations which continues to be a major issue for policymakers, public health experts, and communities across the country [1–3]. The U.S. experiences tens of thousands of firearm-related deaths each year with the latest statistics from 2022 suggesting that there were more than 45,000 firearm-related deaths in the U.S. (including homicides, suicides, and unintentional shootings) [4, 5]. Among all these deaths, suicides by firearms make up the majority proportion (nearly 55% of all gun deaths), and the total cost of firearm violence annually is estimated to be around $500 billion [3–5].

Research on firearm violence in the U.S. has grown in the past few decades. However, most studies and publications have focused on the nature, extent, and distribution of firearm-related deaths in the U.S [5–10]. The burden of non-fatal firearm injuries in the U.S. is a significant and often underrecognized aspect of gun violence, with wide-ranging consequences for individuals, families, communities, and the healthcare system. While fatal gun violence typically garners the most attention, non-fatal injuries also impose a heavy toll [4, 11, 12]. The direct medical costs of treating non-fatal firearm injuries in the U.S. have been estimated to be more than 10 billion per year (i.e. including medical costs, lost productivity, and the costs of law enforcement and criminal justice interventions, etc.) [1, 2, 11–15]. Above all, non-fatal firearm injuries account for a substantial portion of gun-related incidents, far exceeding the number of fatalities. For example, it is estimated that every year, the number of non-fatal firearm injuries is nearly two or more times the number of fatal firearm injuries [1, 3, 12–14]. While the preponderance of studies on firearm violence continues to focus on fatal firearm violence, there is a need for studies on non-fatal firearm injuries given that most studies on non-fatal firearm injuries are limited to certain regions in the U.S, focus on a single year, emphasize younger populations, or do not adequately describe the sociodemographic distribution or determinants of non-fatal firearm injuries [11, 12, 14–17]. The purpose of this investigation was to assess the trends and disparities in non-fatal firearm injury rates among U.S. working-age adults from 2000 to 2021.

## Methods

### Study Participants and Measures

A retrospective analysis of non-fatal firearm injury-related emergency department visits in the U.S. was carried out from years 2000 to 2021. The data for this analysis was derived from the National Electronic Injury Surveillance System-Firearm Injury Surveillance Study (NEISS-FISS) [17–19]. The NEISS-FISS is a nationally representative, stratified probability sample of approximately 100 hospitals with at least six beds and 24-hour emergency services. The dataset documents all injuries where a firearm or gun is mentioned in the emergency department record. In total, the NEISS-FISS data includes 127,287 cases of non-fatal firearm injuries in the U.S. from 1993 to 2021. For the present study, we focused on a subset of 99,933 non-fatal firearm injury cases that occurred between 2000 and 2021. This dataset has a complex survey structure that allows for the production of nationally representative estimates. The data was compiled directly from patient medical records. Additional details on the survey structure and sampling strategy employed in the NEISS-FISS data collection are available in the original data repository [17–19]. Firearm injury is defined as a penetrating injury caused by the discharge of a weapon that uses a power charge to fire a projectile. This includes gunshot wounds sustained from handguns, rifles, shotguns, and BB guns. Additionally, the definition excludes non-penetrating injuries associated with firearms [17, 18]. Working-age adults are defined as those who are 18–64 years of age.

The NEISS-FISS data comprises 47 variables, including demographic characteristics such as sex and race/ethnicity. The data also included variables indicating whether the incident involved alcohol/substance use, crime, verbal argument, or physical fights. The day of the event was categorized as either a weekday (Monday-Friday) or a weekend (Saturday-Sunday). Variables were also included for the place of the event (house/apartment/mobile home; school/sports; street; other property; farmlands; unknown), the intent of the injury (unintentional; assault; self-harm; law enforcement; unknown), and the type of firearm involved (handgun, rifle, shotgun, BB gun, unknown). The method of transport to the emergency department was categorized into a single “EMS” category (combining the original EMS, Ambulance, and Rescue/Fire categories). Similarly, the variable describing the patient’s disposition from the emergency department was recorded into the following categories: Treated, Transferred, Observed, Left against medical advice/without being seen, or dead on arrival [18, 19]. Ethical clearance was not required as we used deidentified publicly available data.

### Statistical Analysis

The analyses were performed using STATA software accounting for the complex sampling structure of the NEISS-FISS data. Raw counts and weighted percentages were calculated to describe the demographic characteristics and injury details. To visualize the overall trends in firearm injury rates by age group from 2000 to 2021, locally estimated scatterplot smoothing (LOESS) regression curves were estimated. Joinpoint regression software (version 5.1.0) was utilized to statistically estimate the number of linear slopes across time that best fit the data. Additionally, a parallel pairwise comparison was conducted to statistically test for slope differences between the 18–25 years, 26–45 years, and 46–64 years age groups. This analytical approach allowed a comprehensive examination of the patterns and trends in non-fatal firearm injuries within the specified age population while accounting for the complex survey design of the NEISS-FISS dataset. Percentages were estimated for each study period (2000–2004, 2005–2009, 2010–2014, 2015–2019, and 2020–2021) and percentage changes were computed for three distinct periods (2000–2009, 2010–2021, and 2000–2021) for each study population characteristic.

**Table 1:** Non-fatal firearm injuries by age in the United States, 2000–2021

	Total (*N*, %)	18–25 (*n*,%)	26–45 (*n*,%)	46–64 (*n*,%)	*P*-value
Unweighted	80,238 (100)	33,787 (40.4)	36,649 (46.2)	9,802 (13.4)	
Weighted number	2,361,627	954,375	1,369,574	377,149	
**Sex**					< 0.001
Male	2,361,319 (85.7)	832,528 (87.2)	929,017 (85.2)	262,058 (82.8)	
Female	337,716 (14.3)	121,740 (12.8)	161,703 (14.8)	54,273 (17.2)	
**Race**					< 0.001
NHW	652,114 (35.7)	217,744 (29.6)	296,243 (35.1)	138,127 (56.2)	
NHB	891,648 (48.8)	390,997 (53.1)	417,789 (49.5)	82,862 (33.7)	
Hispanic	234,652 (12.9)	107,401 (14.6)	108,608 (12.9)	18,643 (7.6)	
Asian	1190 (0.1)	170 (0)	868 (0.1)	152 (0.1)	
AI/AN	1079 (0.1)	226 (0)	772 (0.1)	81 (0.1)	
Others	44,773 (2.5)	19,229 (2.6)	19,699 (2.3)	5,845 (2.4)	
**Involvement**					< 0.001
Argument	146,111 (17.2)	52,930 (16.6)	72,975 (19.2)	20,206 (13.6)	
Crime	286,739 (29.8)	111,995 (30.8)	135,882 (31.5)	38,862 (23.2)	
Alcohol/substance	146,732 (17.7)	55,688 (18.1)	75,746 (20.0)	15,298 (10.5)	
Fight	240,219 (24.7)	91,936 (24.8)	117,207 (26.9)	31,076 (18.8)	
**Day of Event**					< 0.001
Monday-Friday	1,505,452 (63.7)	603,512 (63.2)	693,112 (63.5)	208,828 (66.0)	
Saturday-Sunday	856,175 (36.3)	359,863 (36.8)	397,661 (36.5)	107,651 (34.0)	
**Place of Event**					< 0.001
Home/Apt/mobile home	518,029 (21.9)	184,318 (19.3)	230,921 (21.2)	102,790 (32.5)	
School/sports	62,120 (2.6)	22,484 (2.4)	25,525 (2.3)	14,111 (4.5)	
Street/public	395,188 (16.7)	187,792 (19.7)	173,682 (15.9)	33,714 (10.7)	
Other property	349,836 (14.8)	135,452 (14.2)	169,764 (15.6)	44,620 (14.1)	
Farm	2,864 (0.1)	1,009 (0.1)	1,237 (0.1)	618 (0.2)	
Unknown	1,033,590 (43.8)	423,320 (44.4)	489,644 (44.9)	120,626 (38.1)	
**Intent**					< 0.001
Unintentional	517,711 (21.9)	193,514 (20.3)	219,063 (20.1)	105,135 (33.2)	
Assault	1,617,824 (68.5)	678,968 (71.1)	769,701 (70.6)	169,155 (53.4)	
Self-harm	100,180 (4.2)	24,522 (2.6)	46,168 (4.2)	29,490 (9.3)	
Law enforcement	30,483 (1.2)	9,198 (1.0)	16,657 (1.5)	29,490 (1.5)	
Unknown	95,429 (4.0)	48,173 (5.0)	39,184 (3.6)	8,072 (2.6)	
**Firearm type**					< 0.001
Handgun	593,773 (25.1)	221,861 (23.2)	272,419 (25.0)	99,493 (31.4)	
Rifle	86,035 (3.6)	30,163 (3.2)	36,678 (3.5)	18,194 (5.7)	
Shotgun	79,130 (3.4)	248,33 (2.6)	36,807 (3.4)	18,194 (5.5)	
BB gun	156,887 (6.6)	77,619 (8.1)	57,478 (5.3)	17,490 (6.9)	
Unknown	1,445,802 (61.2)	599,899 (62.9)	686,371 (62.9)	159,532 (50.4)	
**Transported by EMS/Rescue/Ambulance**	1,364,809 (57.8)	542,052 (56.8)	647,678 (59.4)	175,079 (55.3)	< 0.001
**ED disposition**					< 0.001
Treated	1,265,557 (53.6)	518,974 (54.4)	571,571 (52.4)	175,012 (55.3)	
Transferred	94,690 (4.0)	36,783 (3.9)	43,404 (4.0)	14,503 (4.6)	
Hospitalized	835,435 (35.4)	334,959 (35.1)	397,421 (36.4)	103,055 (32.6)	
AMA/LWBS	35,808 (1.5)	12,853 (1.3)	17,808 (1.6)	5,147 (1.6)	
Observation	27,574 (1.2)	10,805 (1.1)	12,558 (1.2)	4,211 (1.3)	
DOA	100,754 (4.3)	39,184 (4.1)	47,155 (4.3)	14,415 (4.6)	

AMA: Against medical advice; LWBS: Left without being seen; DOA: Dead on arrival

**Fig. 1:**
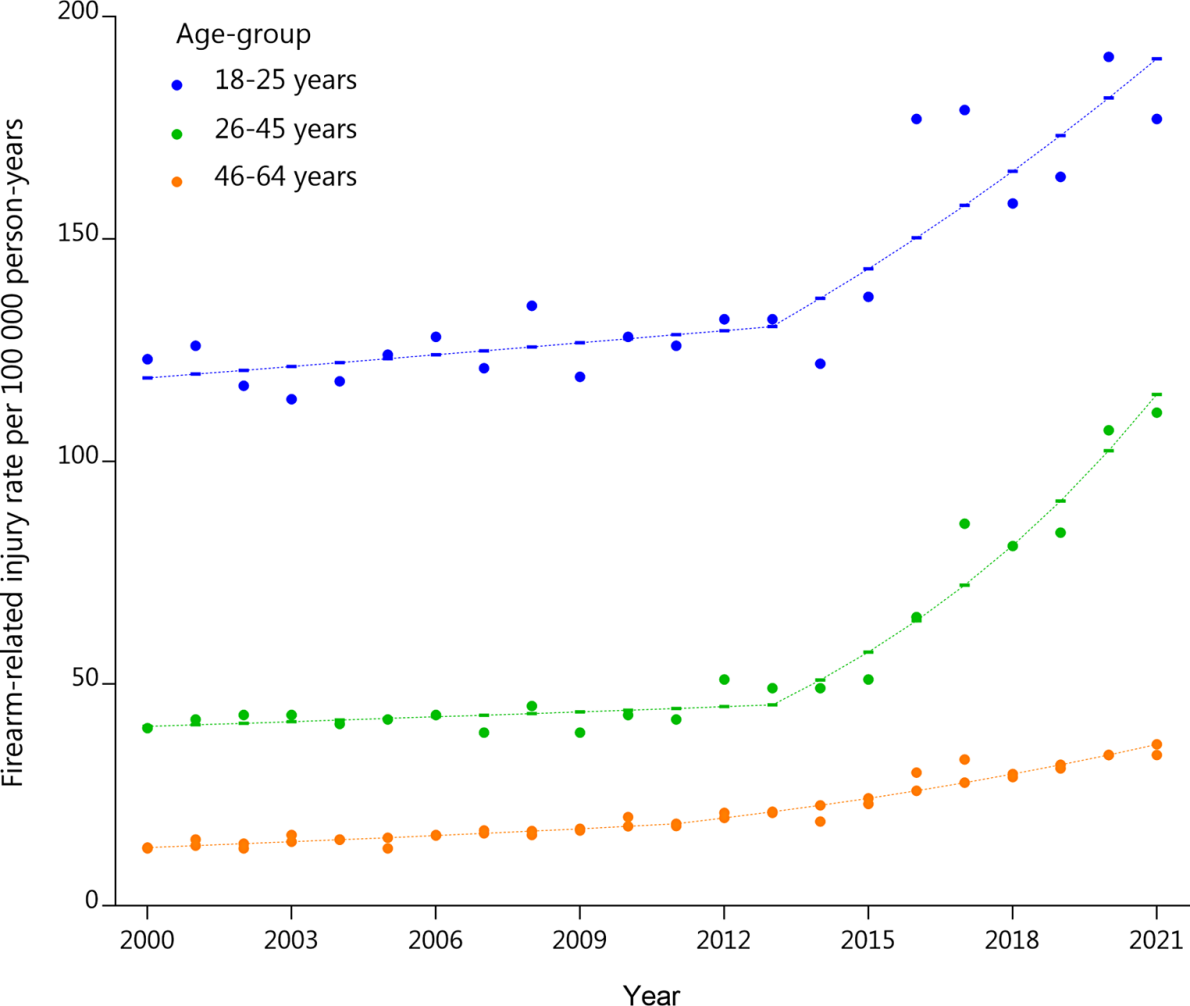
Age-standardized incidence of Firearm-Related Injury Among Individuals Aged 18–64 Years in the USA from 2000–2021. Includes locally estimated scatterplot smoothing regression estimated firearm-related injury rates and 95% CI. Joinpoint regression analysis indicated that the best fitting model includes two linear slopes (one joinpoint) for the 18–25 years trend line, with a significant annual percentage change (APC) observed from 2013 (2013–2021: 4.9%, SE = 2.7, *p* = 0.0004). The best fitting model for the 26–45 years age group also includes two slopes (one joinpoints), with a significant APC of 12.4% (SE = 1.6, *p* < 0.0000001) observed between 2013–2021. For the 45–64 years age group, the best fitting model includes two linear slopes (one joinpoints), with a significant APC of 7.0% (SE = 2.8, *p* = 0.01)

## Results

Table 1 shows the raw counts and weighted percentages of firearm-related injuries in the United States from 2000 to 2021. Overall, there were an estimated 2.9 million non-fatal firearm injuries during the study period, with the majority (85.7%) occurring among the 18–64 age group. Within this age group, males accounted for 85.7% of injuries, while non-Hispanic Blacks had the highest rate at 48.8%. Firearm injuries were commonly associated with crime (29.8%), physical fights (24.7%), alcohol/substance use (17.7%), and verbal arguments (17.2%). Most incidents occurred on weekdays (63.7%) and at unknown locations (43.8%). Assaults were the most prevalent intent (68.5%), followed by unintentional injuries (21.9%). Handguns (25.1%) and unknown firearm types (61.2%) were the weapons most commonly involved. Over half (57.8%) of the patients were transported by EMS/rescue services, with the majority treated and released (53.6%) or hospitalized (35.4%).

When stratified by age, the highest injury rates per 100,000 person-years were found in the 18–25 age group, with significant involvement in crime (30.8%), physical fights (24.8%), alcohol/substance use (18.1%), and verbal arguments (16.6%) (Fig. 1). Incidents mainly occurred on weekdays (63.2%) and at unknown locations (44.4%), with assaults being the most common intent (71.1%), followed by unintentional injuries (20.3%). Joinpoint regression analysis indicated that the best-fitting model for the 18–25 age group included two linear slopes (one joinpoint) with a significant annual percentage change (APC) increase of 4.9% in injury rates per 100,00 person-years from 2013 to 2021 (SE = 2.7, *p* = 0.0004). The 26–45 age group showed similar patterns, with crime (31.5%), physical fights (26.9%), alcohol/substance use (20.0%), and verbal arguments (19.2%) as major determinants and incidents mainly occurring on weekdays (63.5%) at unknown locations (44.9%). For the 26–45 age group, the best-fitting model also included two slopes (one joinpoint), showing a significant APC increase of 12.4% in injury rates per 100,00 person-years from 2013 to 2021 (SE = 1.6, *p* < 0.001). The 46–64 years group had comparable results, with crime (23.2%), physical fights (18.8%), alcohol/substance use (10.5%), and verbal arguments (13.6%) being dominant reasons, and the majority of the incidents occurring on weekdays (66.0%) and at unknown locations (38.1%). The 46–64 age group injury rates per 100,000 person-years exhibited a model with two linear slopes (one joinpoint), with a significant APC increase of 7.0% from 2013 to 2021 (SE = 2.8, *p* = 0.01) (Fig. 1).

**Fig. 2:**
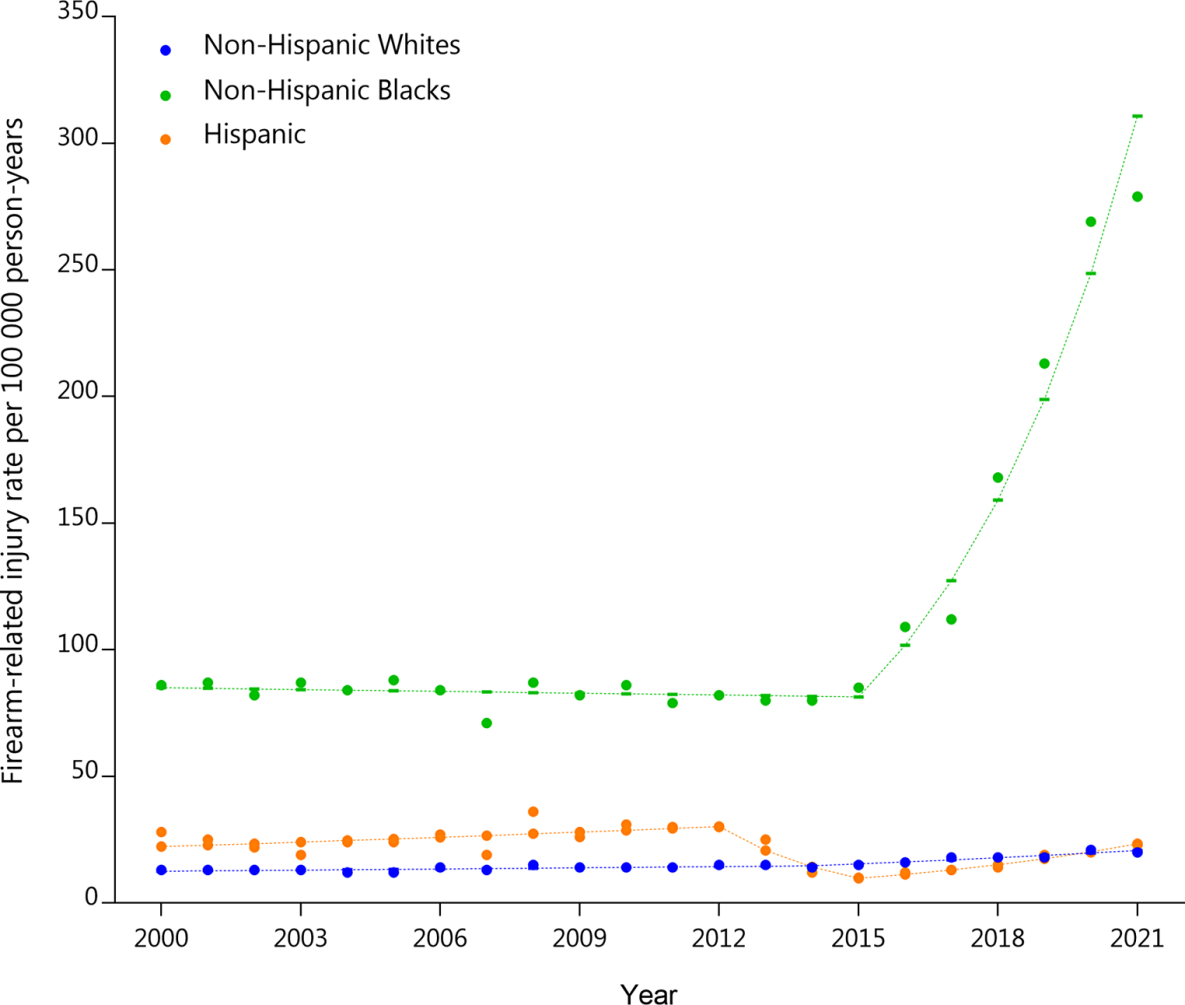
Age-Standardized Incidence of Firearm-Related Injury by Race/Ethnicity in the USA from 2000–2021. Joinpoint regression analysis indicated that the best-fitting model includes two distinct trends (one joinpoint) for the Non-Hispanic White trend line, with a significant APC observed from 2014 to 2021 (APC = 5.0%, SE = 2.4, *p* = 0.0004). For the Non-Hispanic Black population, the best fitting model included one joinpoint, with a sharp increase and a significant APC of 25.0% (SE = 2.5, *p* < 0.000001) observed between 2015 and 2021. For the Hispanic population, the best fitting model includes three linear slopes (two joinpoints), with a significantly reduced APC of -31.3% (SE = 4.8, *p* < 0.0001) from 2012 to 2015, followed by a steep upward trend with an APC of 15.6% (SE = 4.4, *p* < 0.00001) from 2015 to 2021

For racial/ethnic groups, joinpoint regression analysis revealed two distinct trends for non-Hispanic whites on injury rates per 100,000 person-years with a significant APC increase of 5.0% from 2014 to 2021 (SE = 2.4, *p* = 0.0004). The Non-Hispanic Black population showed a sharp increase with one joinpoint, resulting in a significant APC increase of 25.0% in injury rates per 100,00 person-years from 2015 to 2021 (SE = 2.5, *p* < 0.001). The Hispanic population’s trend included three slopes (two joinpoints), with a significant decrease in APC of -31.3% in injury rates per 100,00 person-years from 2012 to 2015 (SE = 4.8, *p* < 0.001), followed by a steep increase of 15.6% in injury rates per 100,00 person-years from 2015 to 2021 (SE = 4.4, *p* < 0.001) (Fig. 2).

**Table 2:** Temporal variations in the proportion of non-fatal firearm injuries among working-age adults, 2000–2021^†^

	2000–2004	2005–2009	2010–2014	2015–2019	2020–2021	% change (2000–2009)	% change (2010–2021)	% change (2000–2021)
**Sex**								
Male	86.8	87.0	86.7	84.6	83.9	-0.23	-3.2	-3.34
Female	13.2	13.0	13.3	15.4	16.1	-1.52	21.06^*^	21.97^*^
**Race**								
NH-White	39.0	38.9	39.5	35.6	25.1	0.25	-36.46^*^	-35.65^*^
NH-Black	44.6	42.0	40.4	53.1	63.4	-5.83	56.94^*^	42.15^*^
Hispanic	13.7	16.6	17.6	8.6	9.0	21.16	-48.83^*^	-34.31^*^
**Age group**								
18–25	44.7	45.3	42.4	37.8	32.8	1.35	-22.65	-26.63
26–45	43.9	42.4	43.4	47.5	54.0	-3.42	24.5^*^	23.01^*^
46–64	11.4	12.3	14.3	14.7	13.2	7.90^*^	-7.70^*^	15.78^*^
**Weekday**								
Mon-Fri	62.9	62.3	63.3	65.1	64.3	-0.96	1.58	2.23
Sat-Sun	37.1	37.7	36.7	34.9	35.7	1.61	-2.72	-3.78
**Firearm type**								
Handgun	31.3	28.6	26.7	21.8	19.0	-8.62	-28.90	-39.30
Rifle	6.6	5.0	3.9	2.2	1.4	-24.25	-64.10	-78.79
Shotgun	5.7	4.6	3.4	2.3	1.5	-19.30	-55.89	-73.69
BB gun	8.5	9.1	7.7	5.2	3.2	7.1*	-54.5	-62.35
Unknown	48.0	52.7	58.3	68.5	74.9	9.8*	28.4*	56.05*
**Involvement**								
Verbal Argument	22.0	16.7	14.7	16.9	17.1	-24.09	16.33	-22.27
Crime	34.6	31.7	32.3	30.0	15.9	-8.40	-50.78	-54.05
Alcohol & Substance use	18.3	8.1	8.1	26.2	25.0	-50.74	208.65	36.62
Physical Fight	28.5	26.7	24.3	24.2	19.6	-6.32	-19.35	-31.23
**Intention**								
Unintended	11.4	22.3	23.6	21.4	18.6	95.62	-21.19	63.16
Assault	23.5	63.8	66.5	73.5	78.0	171.49*	17.30*	231.92*
Self-harm	58.1	4.7	4.8	3.6	2.6	-91.91	-45.84	-95.53
Law enforcement	5.7	1.4	1.5	1.4	0.6	-75.44	-60.0	-89.48
**ED disposition**								
Treated	56.7	55.7	53.7	51.0	52.7	-1.77	-1.86	-7.06
Transferred	5.0	4.9	3.9	3.3	3.5	-2.0	-10.26	-30.0
Hospitalized	31.7	32.1	34.2	37.6	40.3	1.27	17.84	27.3
AMA/LWBS	0.6	0.9	1.8	1.8	2.3	50.0	27.78	283.34*
Observation	0.5	1.1	1.5	1.3	1.3	120	-13.33	160.0
DOA	5.5	5.0	4.9	5.0	0.1	-9.09	-97.75	-98.18
**Unweighted total**	13,702	15,612	16,869	21,737	12,318	-	-	-
**Weighted total** (%)	393,077 (16.6)	421,234 (17.8)	473,894 (20.1)	700,917 (29.7)	372,505 (15.8)	-	-	-

^†^weighted percentages & percentage changes; AMA: Against medical advice; LWBS: Left without being seen; DOA: Death on Arrival. *P_*Trend*_<0.001

Temporal and demographic variations in the proportion of non-fatal firearm injuries among U.S. adults aged 18–64 years from 2000 to 2021 revealed statistically significant changes over time and across different demographic groups (Table 2). The proportion of firearm injuries among males decreased from 86.8% in 2000–2004 to 83.9% in 2020–2021 (3.3% decrease), while the proportion among females increased from 13.2 to 16.1% (21.97% increase) during the same period. Racial/ethnic variations showed that the percentage of injuries among non-Hispanic whites declined from 39.0 to 25.1% (35.65% decrease), while non-Hispanic Blacks experienced an increase from 44.6 to 63.4% (42.15% increase) during the study period. Hispanic individuals saw a decrease in the proportion of injured due to firearms from 13.7 to 9.0% (34.3% decrease). Age-specific trends indicated that the 18–25 age group had a decrease in injury rates from 44.7 to 32.8% (26.63% decrease), whereas the 26–45 age group saw an increase from 43.9 to 54.0% (23% increase) and 46–64 age group had an increase from 11.4 to 13.2% (15.78% increase).

Examination of incident characteristics found a decrease in injuries occurring on weekends from 37.1 to 35.7%, with weekday incidents increasing from 62.9 to 64.3%. The types of firearms involved also changed over time, with handgun-related injuries declining from 31.3 to 19.0% (39.3% decline), rifle-related injuries decreasing from 6.6 to 1.4% (78.79% decline), and shotgun-related injuries shifting from 5.7 to 1.5% (73.69% decline). However, injuries related to unknown firearm types increased statistically significantly by 56.05% Involvement in verbal argument-related firearm injuries decreased from 22.0 to 17.1% (22.27% decline), crime-related firearm injuries decreased from 34.6 to 15.9% (54.05% decline), and physical fight related firearm injuries declined by 31.2%. Firearm injuries related to alcohol/substance use increased by 36.6% from 2000 to 2021. The intent behind the injuries revealed a statistically significant increase in assault-related incidents from 23.5 to 78.0% (231.9%) and an increase in unintended firearm injuries from 11.4 to 18.6% (63.16% increase). Statistically significant decreases were also observed for self-harm (95.5% decline) and law enforcement-related firearm injuries (89.48% decline). Significant increases were also found for hospitalization or individuals leaving against medical advice/without being seen after the firearm injury incident (Table 2).

## Discussion

The number of firearm deaths each year does not adequately represent the impact of firearm trauma in the United States. Non-fatal firearm injuries are more than twice as frequent as fatal injuries [20, 21]. Nonfatal firearm injuries have a variety of costs to the injured, their family and friends, and the community. The costs can include medical care costs, physical disabilities assessed as disability-adjusted life years, lowered quality of life assessed as quality-adjusted life years, psychological trauma (e.g. anxiety, depression, substance abuse, and PTSD), potentially shortened lifespan assessed as years of potential life lost, missed work, and trauma recidivism are some of the more common consequences. The vast majority of firearm injury research has focused on firearm-related fatalities [8, 22, 23]. However, within the past decade, there has been a partial shift in firearm-related research to include non-fatal injuries.

An examination of our findings with recently published firearm injury research indicates we have a high degree of concordance [11, 15, 16]. Our results indicate the majority of nonfatal firearm injuries occur in males (85.7%) and others have found rates of 82.5%, 83.2%, and 87.7% [20, 24, 25]. We found the highest rate of non-fatal firearm injuries was in non-Hispanic Blacks (48.8%) and others found the highest rate in Blacks as well (53.0% and 61.2%) [24, 26]. Based on intent, we found assault was the most prevalent intent (68.5%) followed by unintentional firearm injuries (21.9%). Others found the most common intents were assault injuries (38.9%, 56.2%, and 70.4%) followed by unintentional injuries (12.3%, 28.1%, 36.9%) [20, 24, 26, 27]. The type of firearm most frequently used in our study was handguns and this was confirmed by other studies as well. Disparities in findings across the various studies are most likely due to the use of different databases, age ranges of the injured included, variations in the time frames of the studies, and different statistical analyses of the data [14–16, 25–27].

Certain results from this study need further exploration. For example, the highest increase in the proportion of firearm injuries was noted among females and it is unclear if there is a connection with the fact that gun ownership among women in the U.S has increased significantly over the past two decades and women are now the fastest-growing group of gun owners in the country [28–33]. For firearm types, the highest increase across the study period was noted for the “unknown” category indicating a need for better and more specific data collection and coding in such national injury databases (i.e. NEISS) to inform prevention practices [33–36]. Furthermore, the significant and only increase in involvement type for non-fatal firearm injury incidents across the study period was noted for alcohol and substance use; this underscores the need for interventions that address a variety of risk behaviors among those prone to firearm violence victimization. Finally, the most marked increase across the study period of 2000–2021 was noted for unintended firearm injuries (63.16% increase) or injuries due to assaults (231.9%). Again, unintended injuries relate to weaker gun laws and the widespread availability of firearms while assaults are a part of the larger community firearm violence problem across the nation [29–33]. Interestingly and in contrast, there was a marked decline in self-harm or law enforcement-induced non-fatal firearm injuries. This could be because the use of firearms for self-harm or during violent encounters with law enforcement may result in higher lethality and not just injuries [6–9].

Attempts to reduce firearm morbidity and mortality have focused primarily on state and federal policies. The three areas of firearm policies that have shown the most promise in reducing firearm injuries are background checks of purchasers, waiting periods for purchasing a firearm, and extreme risk protection orders [28]. Background checks for firearm purchases are intended to prevent purchases by individuals (e.g. felons, some mentally ill, those convicted of domestic violence, etc.) who likely present a danger to themselves or others, possibly reducing firearm violence [28]. The Brady Handgun Violence Prevention Act of 1994 created federal requirements for licensed firearm dealers to conduct background checks on purchasers of firearms with the FBI’s National Instant Criminal Background Check System (NICS). However, background checks were not required for sales at gun shows, other private sales, and personal gifting of firearms. These loopholes in the background check system led to several states creating “universal background checks”. Research indicates that universal background checks are associated with lower firearm suicides and homicides [29]. A reduction in firearm mortality could be a harbinger of reduced nonfatal firearm injuries as well. Also, some scholars have reported that states with stronger background check laws have reduced rates of total and unintentional nonfatal firearm injuries, suggesting that legislative measures can effectively mitigate firearm-related harm [30].

Waiting periods to purchase firearms are intended to stop impulsive acts of firearm suicide and anger-directed firearm assaults by creating a “cooling off” period. Also waiting periods provide law enforcement the opportunity to assess whether the firearm buyer is a possible straw purchaser (i.e. a legal firearm purchaser who is purchasing for a prohibited firearm purchaser) [28]. As of January 2024, six states and the District of Columbia have waiting periods for all firearm purchasers, 4 states for certain types of firearms (e.g. handguns), and some states have licensing laws (e.g. permits to purchase) that create their waiting periods. State variations and waiting periods range from 3 to 14 days [28]. Research indicates that waiting periods may reduce firearm suicides, but there is limited evidence that firearm homicides are also reduced. Interestingly the most effective time frame for reducing firearm mortality was 2–7 days [28]. This time frame may also be associated with reduced non-fatal firearm injuries. Evidence is lacking for waiting periods affecting unintentional firearm injuries. In contrast, Extreme Risk Protection Order (ERPO) laws, also known as “red flag laws”, exist in 21 states (with state variations) and the District of Columbia [28]. No federal ERPO exists, and these laws create a means for family members, law enforcement officers, and rarely physicians to seek through a petition to civil courts, to temporarily have a firearm removed from individuals considered to be at risk of shooting themselves or others. Also, ERPOs temporarily ban the involved from purchasing a firearm. A study of ERPOs in four states estimated that for every 17–23 ERPOs filed, one suicide could be prevented [28–32].

The aforementioned three policies may modestly reduce firearm injuries if they are fully implemented and enforced. However, the existing evidence does not indicate that any group of firearm policies will substantially (> 50%) reduce injuries. Also, the success of these laws depends on states enforcing these laws, reporting of individuals who should be denied access to firearms, compliance of firearm dealers with the laws, how truthful purchasers are in completing forms to purchase a firearm, and state sociodemographic and political climate. It should also be noted that the ubiquitous availability of firearms in the U.S. greatly reduces the potential for prevention of firearm injuries and it is unlikely that the firearm injury burden will decline while millions of firearms are added each year to the US population arsenal [33]. It may be that for the foreseeable future public health professionals will need to spend more effort in focusing firearm injury research on the environments that result in firearm injuries [34, 35]. Also, improving national data collection on gun violence can help policymakers make informed decisions regarding which interventions work best to reduce firearm-related morbidity and mortality [35, 36].

Firearm violence (e.g. non-fatal injuries) is a peculiar problem for urban, inner-city, low-income, and minority populations in the U.S [34–36]. Based on this observation and the results of our analysis, other interventions that have been discussed but may lack rigorous evidence of effectiveness warrant some discussion [37–49]. These interventions can be implemented at the community or healthcare facility level. First, community-based violence interruption programs are geared towards interrupting the cycle of violence by mediating conflicts before they escalate into shootings. They often involve “violence interrupters” who are individuals from the community, sometimes with backgrounds in gangs or other violence-prone groups, who can diffuse tensions and encourage peaceful resolution (e.g. Cure Violence, a program that focuses on interrupting transmission of violent behavior by identifying and intervening with individuals at high risk of committing or being victims of violence; or the Operation Peacemaker Fellowship, a program that trains individuals who have been involved in violence to intervene in conflicts, provide mentoring, and connect people to services) [37, 38]. Second, enhanced law enforcement strategies may play a crucial role in reducing non-fatal firearm violence through targeted interventions (e.g. Focused Deterrence that involves identifying individuals or groups most at risk of violent behavior and offering a combination of support services alongside the threat of serious legal consequences if they engage in violence; Hot Spots Policing that focuses police resources on areas with the highest incidence of violence using data-driven strategies to pinpoint where interventions may be most needed) [39, 40]. Third, community public health approaches also include education campaigns (e.g. informing individuals about the risks of gun ownership, safe storage and disposal of firearms, and the dangers of impulsive or heated confrontations escalating into gun violence) and mental health services (e.g. providing better access to mental health care, especially for individuals with anger management issues, severe trauma, or untreated psychiatric conditions, can help prevent violent outcomes) [41, 42]. Fourth, economic development and neighborhood investment may help as investing in these communities can help reduce violence by addressing underlying economic factors that contribute to crime (e.g. creating opportunities for employment and economic mobility can reduce the incentive for individuals to engage in criminal activity; community policing and trust-building between law enforcement and communities to help prevent violence and improve cooperation between residents and authorities) [35, 43, 44]. Fifth, technological solutions where new technologies may help prevent firearm violence or assist law enforcement in identifying potential threats (e.g. SmartGuns, these firearms are equipped with technology that can prevent unauthorized use, such as requiring biometric identification to operate firearms; ShotSpotter, technology that uses sensors to detect gunfire in urban areas and alert law enforcement quickly, allowing for faster responses to gun violence incidents) [36, 45, 46]. Sixth, and finally, there is a continuing discussion on the role of clinicians and healthcare facility-based interventions to reduce firearm injuries. These interventions and recommendations are frequently geared towards the provision of anticipatory guidance to patients on firearm safety, screening and risk assessment for firearm injuries, improving mental health care and substance use prevention services, collaboration with community organizations and healthcare professional advocacy, trauma care and prevention of future firearm violence victimization, medical student training on firearm violence prevention, and data collection and research on firearm violence [47–49]. Again, it should be noted that the aforementioned six avenues for intervention are often lacking in evidence for how effective they are in preventing non-fatal firearm injuries or are found to be effective in limited settings. Given the multifactorial nature of firearm violence and the multitude of determinants of non-fatal firearm violence, there is a need for continued research in this area spanning from better data collection to designing interventions for the reduction of such injuries [34–36, 50].

Our study has a variety of potential limitations. Our findings may be limited by the absence of physician office visits for firearm injuries and the injuries in which health care was not sought and the intentionality of nonfatal firearm injuries often relies on the injured’ s self-reports. There may be occasions where the injured may want to not admit the real intention (e.g. suicide or criminal activity) resulting in misclassification of intentions. The NEISS database did not contain numerous demographic variables such as education, economic status, employment status, and state-specific data that could have assisted with a more granular assessment of working-age adults’ non-fatal firearm injuries. Our findings may be limited by inaccuracies in emergency room coding of firearm incidents and racial and ethnic misclassifications. Finally, in several categories of information regarding patients, there were large numbers of unidentified information (e.g. firearm type, place of event, etc.) that could have influenced our findings.

## Conclusions

Non-fatal firearm injuries in working-age U.S. adults are most commonly associated with crimes, fights, and substance abuse. Over 90% of firearm injuries are from assaults and unintentional injuries and primarily occur in younger males. To ameliorate the basis of nonfatal and fatal firearm injuries public health professionals need additional resources to assess not only policies related to firearms but to also expand their research efforts into the psychosocial elements of society that form the substrate of many of our health problems. Also, a multifaceted approach that includes legislative measures, community interventions, and educational programs is essential for effectively reducing non-fatal firearm injuries in the U.S. The evidence supports the notion that comprehensive strategies can lead to significant reductions in firearm-related harm, particularly when they are tailored to the specific needs of communities and individuals at risk.
